# Addressing COVID-19 Vaccine Hesitancy and Uptake Among African Immigrants: Lessons from a Community-Based Outreach Program

**DOI:** 10.1007/s40615-024-01947-9

**Published:** 2024-03-05

**Authors:** Emmanuel F. Koku, Nettie Johnson-Yengbeh, Ava Muhr

**Affiliations:** 1 Department of Sociology, Drexel University, 3201 Arch Street, Room 288, Philadelphia, PA 19104 USA; 2 Health Department, African Cultural Alliance of North America (ACANA), 5530 Chester Ave, Philadelphia, PA 19143 USA; 3 School of Social and Political Science, University of Edinburgh, 15a George Square, EH8 9LD Edinburgh, UK

**Keywords:** African immigrants, COVID-19, Vaccine hesitancy, Community health outreach, Community-based organizations, Mixed-methods

## Abstract

In 2021, the African Cultural Alliance of North America (ACANA) implemented a community-based vaccine education and outreach program to decrease hesitancy and increase COVID-19 vaccine uptake among African immigrants in Philadelphia. The program had three components: (1) tailored messaging on the benefits of vaccines by trusted community *health navigators* in familiar languages/dialects, (2) use of *educational/tabling* events, and (3) establishment of a *vaccine clinic* in community settings. Using secondary data analysis, in-depth interviews, focus group discussions and a self-administered survey, we explored (i) the impact and effectiveness of the outreach program and extent of vaccine uptake, (ii) African immigrants’ beliefs about the COVID-19 pandemic and the vaccine, and (iii) barriers and facilitators of vaccine knowledge, uptake, and hesitancy. Our analysis showed that ACANA’s outreach program was effective in addressing several cultural, logistic, and systematic barriers to vaccine uptake. The program distributed 2000 educational/informational flyers, reached 3000 community members via social media campaigns, and an additional 2320 through other person-to-person outreach events. The program was effective and resulted in the vaccination of 1265 community members over the course of the outreach. The impact of this outreach underscores the critical role of community-based organizations in addressing COVID-19 vaccine hesitancy and increasing vaccine uptake in underserved and minority communities. The paper concludes with suggestions and recommendations for using community-based outreach programs to increase COVID-19 vaccine uptake and decrease hesitancy.

**Supplementary Information:**

The online version contains supplementary material available at 10.1007/s40615-024-01947-9.

## Introduction

The COVID-19 pandemic created challenges and opportunities for health systems globally. It stretched the preparedness capabilities of public health authorities, exposed existing health inequalities and increased mortality in vulnerable populations [1]. At the same time, it also provided opportunities to restructure public health systems to better respond to pandemics and stem the spread of diseases [2]. Fortunately, the successful implementation of COVID-19 testing protocols and the subsequent development of vaccines have tremendously slowed COVID-19 infections and comorbidities such as cardiovascular, kidney, and neurodegenerative diseases [3].

Despite these successes, there are glaring ethnic, racial, and socio-economic disparities in the risk and testing for COVID-19 [4–6]. In particular, Black and Brown communities in the United States (US) have borne the brunt of the COVID-19 epidemic: they have experienced higher rates of COVID-19 infections and related comorbidities, hospitalizations, deaths, lower vaccination coverage, and higher hesitancy compared to non-Hispanic Whites [7–10]. Within Black and other racial minority groups in the US, preexisting structural inequalities further exacerbated disease burden, while incorrect beliefs, misinformation, lack of trust in governments and pharmaceutical companies, conspiracy theories, vaccine denialism, and medical mistrust have been linked to increased COVID-19 vaccine hesitancy and slower uptake [11–19]. Ever since the 72^nd^ World Health Assembly in 2019, the World Health Organization has emphasized the need to pay closer attention to the health needs and priorities of refugee and migrant populations [20]. Consequently, it is critical to focus specifically on Black immigrants in the US due to the particular barriers they face as a result of their racial background and immigration status [21].

Black migrants, especially those from sub-Saharan Africa, are a growing component of the Black population in the US [22]. Roughly two million sub-Saharan African immigrants in the country reside in selected states (New York, Florida, Georgia, and Pennsylvania) most impacted by the COVID-19 epidemic [23, 24]. Like migrant groups, Black immigrants face numerous structural barriers to care [25–27]. Acculturative stressors (language limitations, legal status concerns), access barriers (insurance coverage, transportation), lack of knowledge of new health systems, and poor health literacy affect their access and utilization of COVID-19 prevention and treatment services [28–30]. Moreover, broader social determinants, including poverty, and residential living conditions that do not allow social distancing or quarantining increase their risk for and exposure to COVID-19. In addition, some African immigrant’s occupational choices (especially the tendency to work in occupations regarded as “essential” or positions with limited remote-work or paid leave options) put them at the epicenter of the epidemic [28, 29, 31–33].

Despite these risks and potential exposure, to our knowledge, only a few studies have examined vaccine acceptability, uptake, and hesitancy among African immigrants in the US [22, 30, 34] and elsewhere [28, 35]. Even fewer studies have examined how these populations responded to the epidemic and addressed vaccine hesitancy and uptake [36], despite increasing evidence pointing to the acceptability of such outreach in health preventive programs in African immigrant and other minority communities [37–41]. To fill some of these gaps, this paper presents the results of a community-based vaccine education and mobilization program implemented by the African Cultural Alliance of North America (ACANA), in partnership with the Philadelphia Department of Public Health (PDPH), to understand and address COVID-19 vaccine hesitancy and uptake among African immigrants living in the Philadelphia region. The paper explores (i) the impact and effectiveness of the outreach program and extent of vaccine uptake, (ii) African immigrants’ beliefs about the COVID-19 pandemic and the vaccine, and (iii) barriers and facilitators of vaccine knowledge, uptake, and hesitancy.

## Method

### Participants

**Fig. 1:**
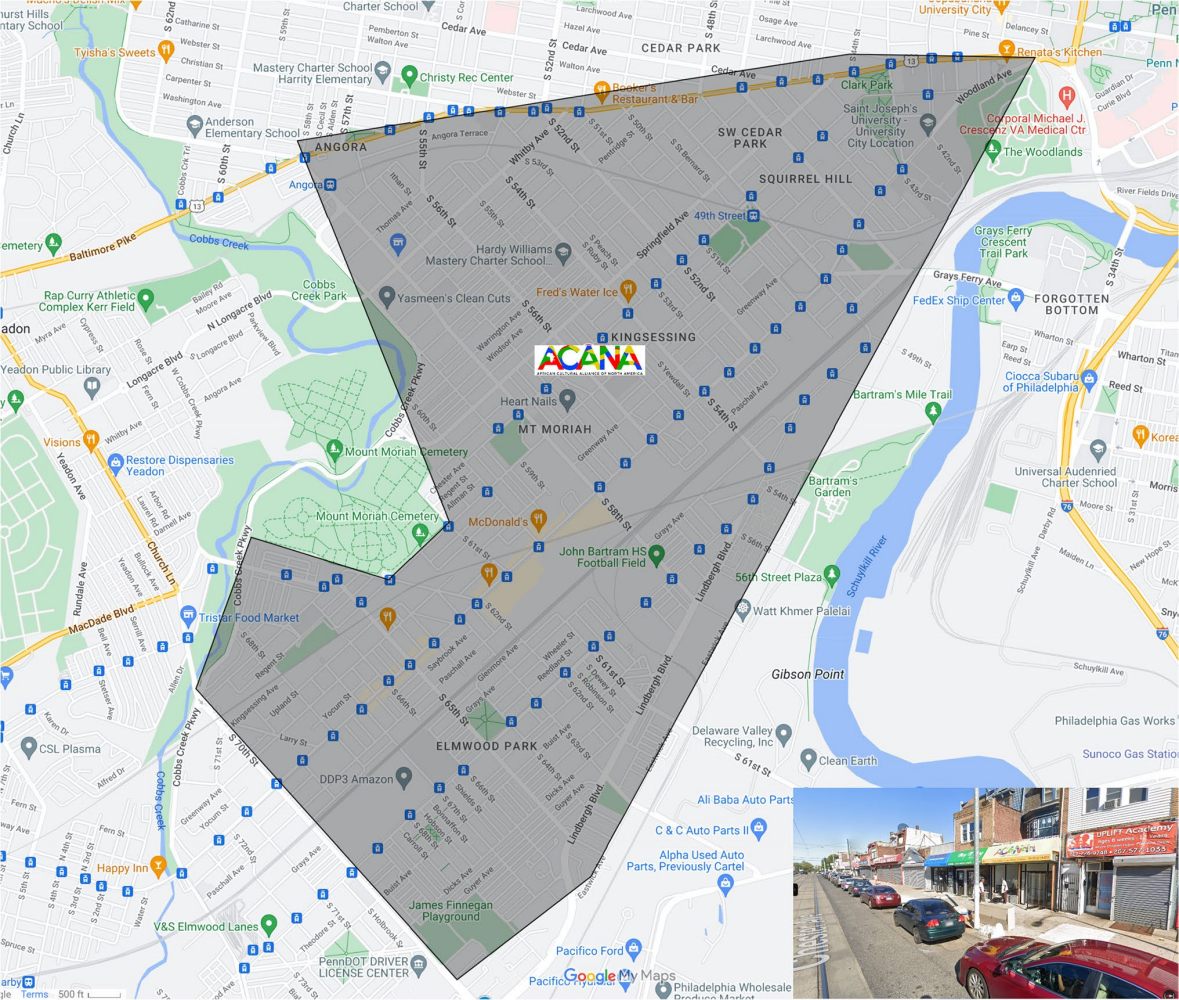
Study site

African Cultural Alliance of North America Inc. (ACANA) is a 501 (c)(3) non-profit organization located in Southwest Philadelphia (see Fig. 1). ACANA was founded in 1999 by a group of African musicians who sought to establish themselves in the US to promote arts and culture. ACANA soon expanded its original mandate to include a range of social services to meet the needs of recently resettled African refugees and asylees who were fleeing civil wars from West Africa.

Today, ACANA is a trusted organization with unique expertise and offers culturally sensitive programs to asylum seekers, refugees, and other immigrants in Philadelphia, in partnership with stakeholders such as the Philadelphia Department of Public Health, educational institutions, medical foundations, and other community-based organizations (CBOs). Some of these programs include public health education and health screenings(against HIV, mental health stigma, female genital cutting, and cardiovascular and other chronic diseases), comprehensive legal and immigration services, English language classes, financial counseling, the arts, and cultural entertainment.

Study participants are drawn from this larger community of African immigrants living in Philadelphia and served by ACANA. In recent years, Philadelphia has become one of the main destinations of African immigrants, with an estimated population of 40 to 50,000 residing in the region [42]. More than half of all African immigrants in the Philadelphia area come from West African countries, with the largest concentrations from Liberia, Ghana, Nigeria, Sierra Leone, and Togo. Most of these immigrants reside in dense low-income housing or large multigenerational households in Southwest Philadelphia and other suburban communities [43]. Close to a third of the sub-population within ACANA’s target communities live below the federal poverty line [43, 44]. They have little to no insurance coverage and face several structural barriers that affect their use of health services [21, 45, 46].

### Data Collection

The study used a mixed mixed-methods design, comprising secondary data/documentary analysis, in-depth interviews and focus group discussions, and a self-administered survey.

#### *Secondary Data and Documentary Analysis:*

The research team collected and reviewed reports from ACANA’s records about its COVID testing and vaccine campaign. These data included types/number of community outreach events held, number of attendees at each event, number of vaccine clinics held per month, the total number of people tested and vaccinated at each event, socio-demographic characteristics of those tested and vaccinated. Among others, this retrospective data helped us evaluate the impact and effectiveness of the outreach program.

#### *Survey of Clients:*

An anonymous survey was administered to a convenience sample of 100 clients who attended ACANA events in the fall of 2022. The survey was paper-based and self-administered, although two of the co-authors were available to answer any questions or concerns about the survey, as well as connect the individual with further translation assistance when required. The survey collected data on clients' self-reported COVID-19 testing and vaccine history, perceptions of, knowledge about COVID-19, reasons for vaccine hesitancy, and suggestions for increasing uptake (see *Appendix*).

#### *Focus Groups and Interviews:*

In addition to the survey, ACANA conducted focus group discussions with 12 community members and 6 health navigators, and in-depth interviews with 3 clinical staff to explore the efficacy of its efforts during the fall of 2022, as the organization responded vaccine hesitancy and rising reinfections from new/resistant COVID-19 strains. The interviews were semi-structured and were conducted in person or over the phone, depending on participants’ preferences, by two members of the research team. The focus group discussions were held onsite (at ACANA) and were facilitated by a social worker and the health intern. The focus group discussions were held in English, but questions were translated into French and Mandingo, a West African language, when required. The focus groups were homogeneous: all participants were African immigrants who resided in Southwest Philadelphia and were affiliated with or have received services from ACANA. They all shared similar experiences and challenges with COVID-19 pandemic and vaccine rollout. 

These interviews and focus group discussions covered a range of topics, including community members’ beliefs about, understandings of, and attitudes toward COVID-19 testing and vaccination; structural and clinical barriers and facilitators of access, testing, and vaccine uptake; effectiveness of strategies used by health navigators and clinical teams to promote testing and vaccination in community settings(see *Appendix*). Participants were not provided any monetary incentives, but were given souvenirs (pens, health promotional materials) for completing the study. The research team developed the survey instrument, focus group and in-depth interview guides based on the extant literature and insights from their experience working with community-based health outreach programs.

### Data Processing and Analysis

**Fig. 2:**
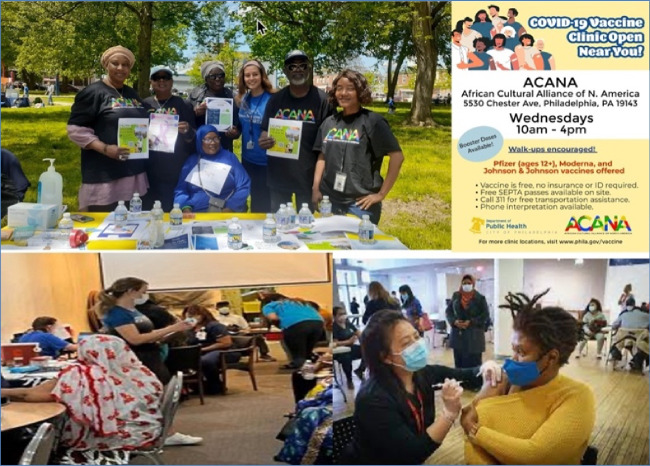
Health navigators and vaccine clinics

Data from the in-depth interviews and focus group discussions were audio-recorded and transcribed with identifying information removed. Recordings in French or other African languages were translated into English and transcribed by a trained bilingual staff from ACANA. To increase credibility of our findings [47], we aimed at analyst triangulation, by jointly participating in the review of transcripts, development, and analysis of themes. A research assistant checked each transcript for accuracy and, with the assistance of the primary research/author, they reviewed them, and summarized their contents [48]. Using thematic analysis [49], each of us (co-authors) independently and separately reviewed the interview transcripts, then coded and grouped them according to themes, guided by our literature review. We then discussed and compared our respective analyses of the narratives, resolved any discrepancies by consensus, and agreed on a final set of themes (and sub-themes) that emerged from the data. We also validated these themes through member checking with selected participants [50]. Finally, we selected representative quotes of each theme to be reported in our findings. Socio-demographic characteristics of participants are presented in Table 1 (*Panel A*). Data from the surveys was de-identified and processed, and descriptive statistics were generated using Excel. Findings from the survey are presented in Table 1 (*Panel B*) and Fig. 2. Data collection for each component of the study (surveys, focus group discussions and in-depth interviews) lasted 30 to 60 min on average, and occurred at ACANA Offices and event sites. The study was reviewed and approved by [NAME] University’s Institutional Review Board.

**Table 1:** Socio-demographic description of study participants

Panel A: Focus group and in-depth interview participants							Panel B: Survey respondents	
Name	Gender	Age range (years)	Educational level	Preferred language	Country of origin	Zip code	Socio-demographic variables	% (*N* = 100)
*Community members*								
CP1	Female	20–30	High school	English	Liberia	19143	*Gender*	
CP2	Male	40–50	High school	English/French	Liberia	19153	Female	69.0
CP3	Male	40–50	Elementary school	English	Nigeria	19142	Male	31.0
CP4	Female	30–40	College	Mandingo	Guinea	19142		
CP5	Male	50–60	High school	French	Mali	19153	*Age*	
CP6	Female	50–60	High school	English	Liberia	19136	Under 25 years	19.0
CP7	Female	40–50	College	French	Burkina Faso	19143	25–44 years	40.0
CP8	Female	50–60	High school	English	Liberia	19154	45–64 years	28.0
CP9	Male	40–50	Elementary school	English	Ghana	19131	65+	13.0
CP10	Female	30–40	College	French/Mandingo	Liberia	19143		
CP11	Male	30–40	Elementary school	English	Mali	19154	*Region of origin*	
CP12	Female	30–40	High school	French	Guinea	19150	West Africa	77.0
							East Africa	16.0
*Health navigators*							North Africa	7.0
HN1	Female	30–40	High school	English	Liberia	19153		
HN2	Female	40–50	High school	English/Vai	Liberia	19142	*Religious affiliation*	
HN3	Female	30–40	College	French	Mauritania	19142	Christian	59.0
HN4	Female	30–40	College	French	Guinea	19153	Muslim	29.0
HN5	Female	40–50	High school	English/Mandingo	Sierra Leone	19143	Traditional	12.0
HN6	Male	40–50	College	English/Mano	Liberia	19153		
							*Years in USA*	
*Clinical staff*							< 5 years	10.0
CS1	Female	30–40	College	French	Guinea	19143	6–10 years	15.0
CS2	Female	30–40	College	English	Liberia	19143	11–20 years	46.0
CS3	Female	30–40	College	English	Liberia	19143	> 20 years	24.0
							Not answered	5.0

#### Findings

We present our findings below. The section “Socio-demographic Description of Study Participants” presents background information about study participants. The next section, “Outreach Strategies” outlines the community outreach programs and their reach/effectiveness, using secondary data and results from the survey. In the section “Understanding Vaccine Uptake Barriers and Hesitancy,” we present the three themes developed from the in-depth interviews and focus groups to help us understand and situate vaccine hesitancy. The final section “Strategies to Increase Vaccine Uptake” presents recommendations and lessons learned from the outreach.

### Socio-demographic Description of Study Participants

Table 1 presents socio-demographic background information of study participants. Twelve community members (7 women and 5 men, age range 25–60 years) participated in the focus groups. All six health navigators (except one) and three clinical staff were women (see Table 1 (*Panel A*)). Most participants have attained at least a high school level of education and were fluent in English, French Creole, and Mandingo.

Reflective of the ACANA’s target population, many of our participants are from West Africa (Liberia, Sierra Leone, Ghana, Guinea, Nigeria); a few were from Mali, Burkina Faso, and Mauritania (North Africa). Survey respondents are similar in composition (see Table 1 (*Panel B*)): the majority (70%) were female and were between 25 and 64 years old. Most identified as either Christian (59%) or Muslim (29%). The typical respondent has been in the US between 10 and 20 years. About a quarter (25%) each were newer (i.e., have been in the US for less than 10 years) or long-term residents (in the country for over 20 years).

### Outreach Strategies

ACANA launched its COVID-19 testing and vaccination outreach program in January 2021. It began with testing and continued with the vaccine outreach campaign until 2023 (when its clinic, discussed below, closed). This paper presents data from the first 2 years of the campaign (January 2021 to December 2022).

**Fig. 3:**
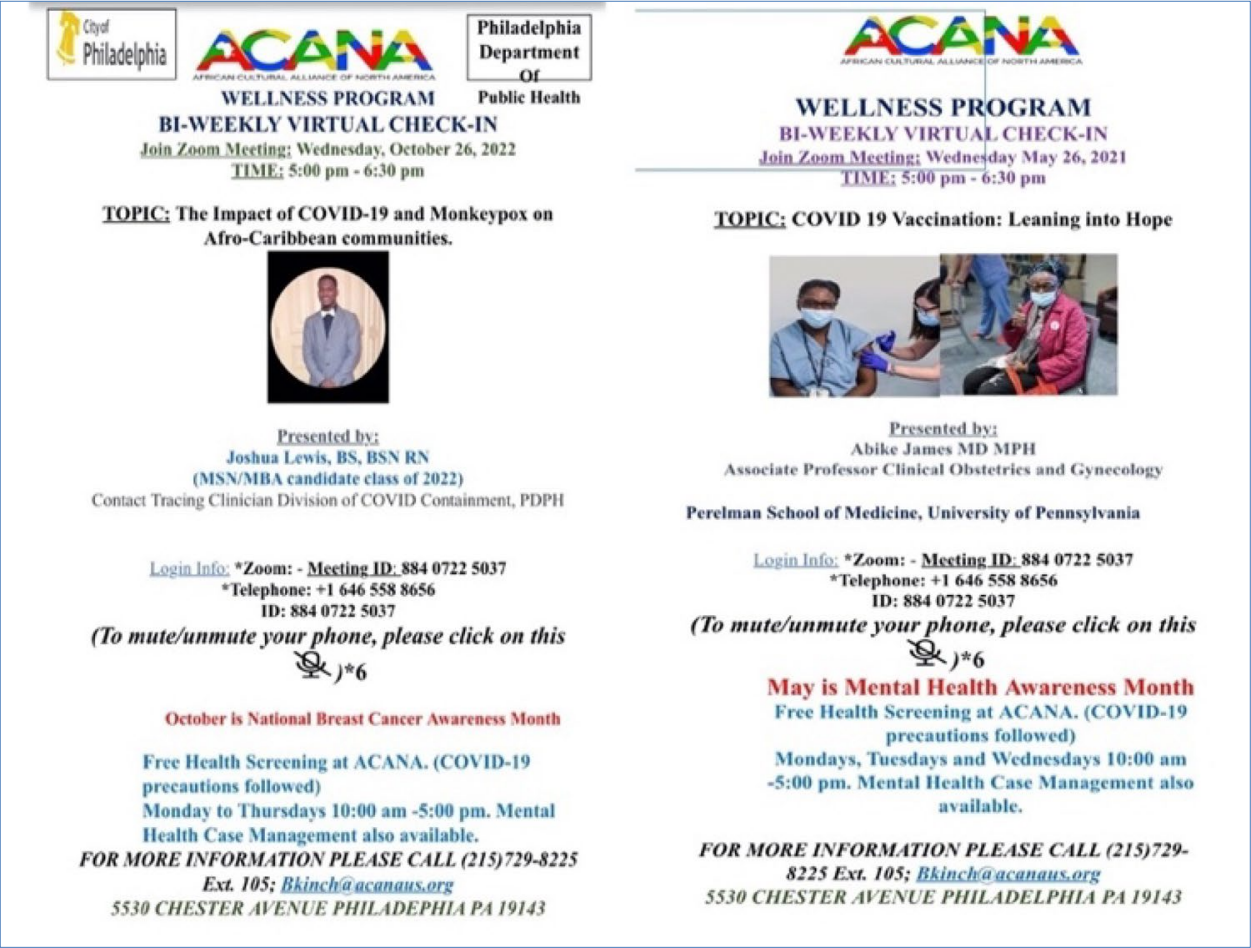
Wellness meetings notices

**Fig. 4:**
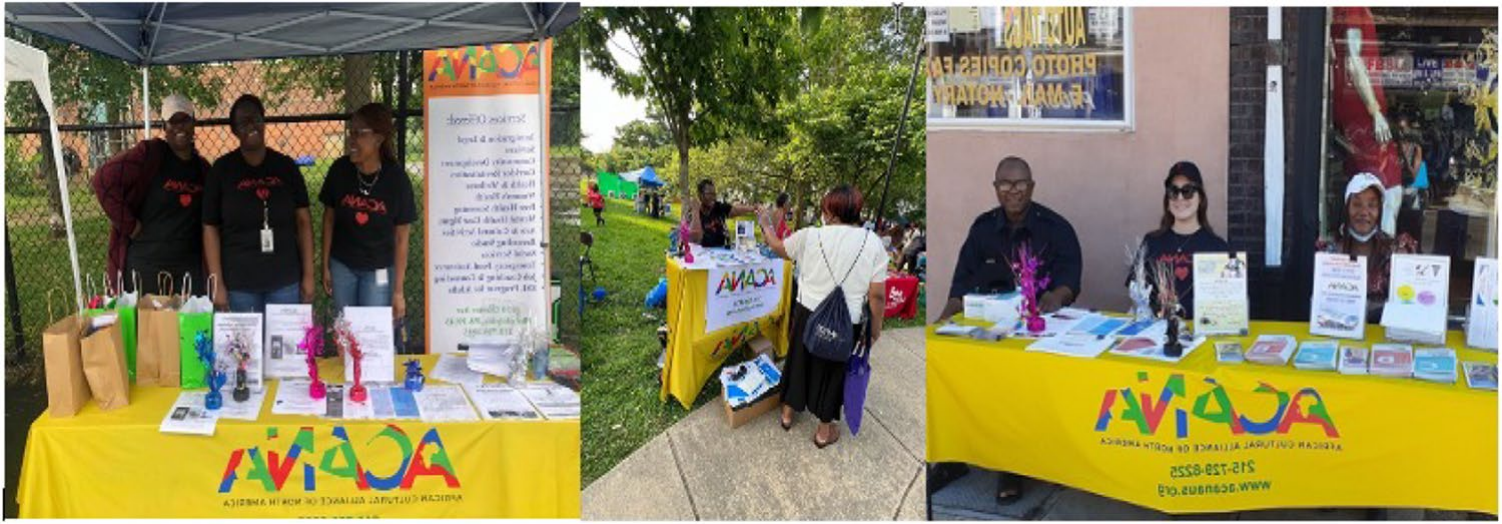
Tabling events

The focal point for this outreach is ACANA’s *vaccine clinic*, operated in collaboration with the Philadelphia Department of Public Health (PDPH) (see Fig. 2). Strategically located on a busy business corridor, the clinic was more accessible to the target community than other vaccine locations in the city. To link community members there, and provide culturally tailored services, ACANA recruited and trained six *health navigators*, who were fluent in several West African languages/dialects (i.e., French, Wolof, Mandingo, Kpelle) spoken in the community (see Fig. 2). These health navigators also helped coordinate key outreach activities such as tabling events, door-to-door canvassing, and wellness meetings. For years, ACANA has used *wellness meetings* as a medium to interact with and educate community members on a range of health topics (such mental health, cardiovascular health, healthy living and physical activity, HIV/AIDS, effects of female genital cutting, and cancer), address health misinformation, and promote health and wellness (see Fig. 3). Twice per month, ACANA’s health navigators set up *Tabling Events* in various community locations and partnered with churches, mosques, local businesses, and stores to disseminate COVID-19 education materials, masks, and protective equipment (see Fig. 4).

**Fig. 5:**
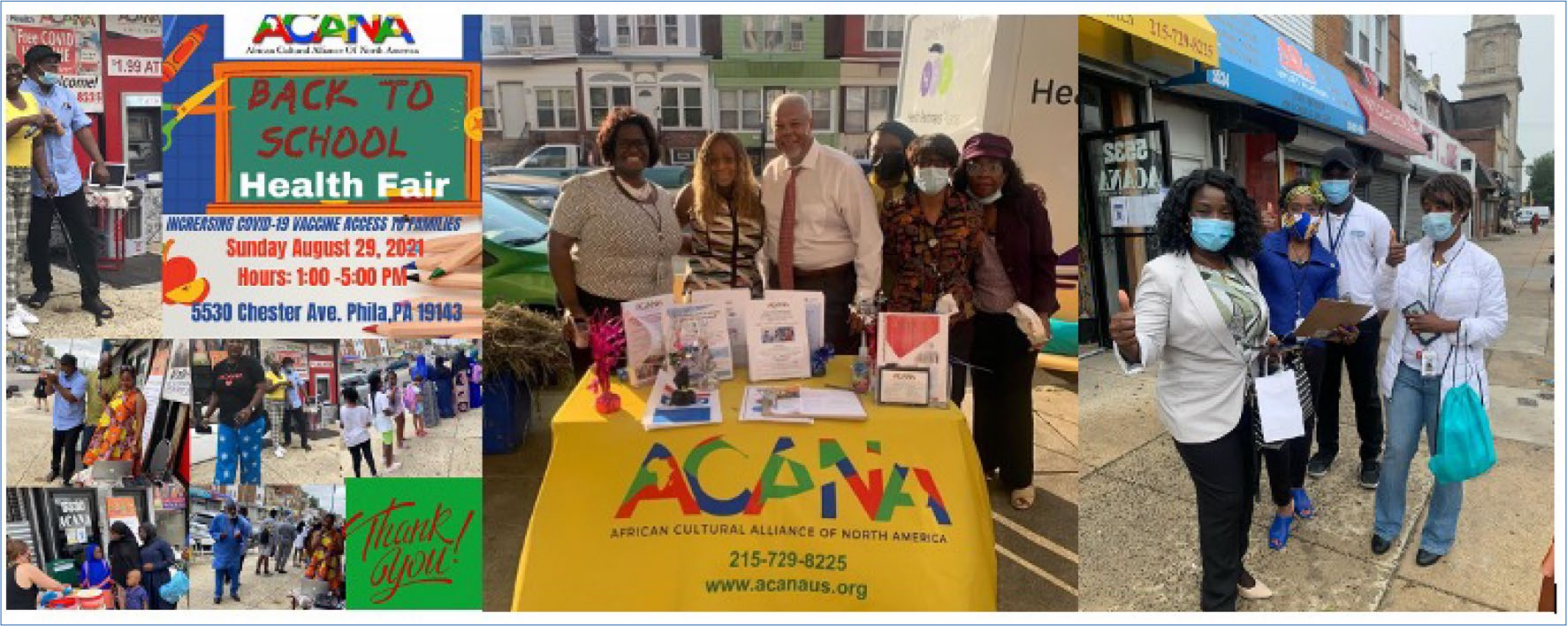
Community walks and door-to-door canvasing

Using input from community members, ACANA designed *flyers and social media messages* to educate and address local misinformation about the disease and circulated them on poster boards, radio, Facebook, and WhatsApp, as well as at the tabling events. These messages were translated into and/or delivered in West African languages and dialects in order for it to be accessible to the population [51, 52] (Fig. 5).

During all these events, health navigators explored community members’ vaccine knowledge and barriers to uptake, reinforced the importance of masking, testing, and vaccination, addressed misinformation, and referred or accompanied community members to vaccine clinics, when needed.

### Outreach Effectiveness: Number Reached

**Fig. 6:**
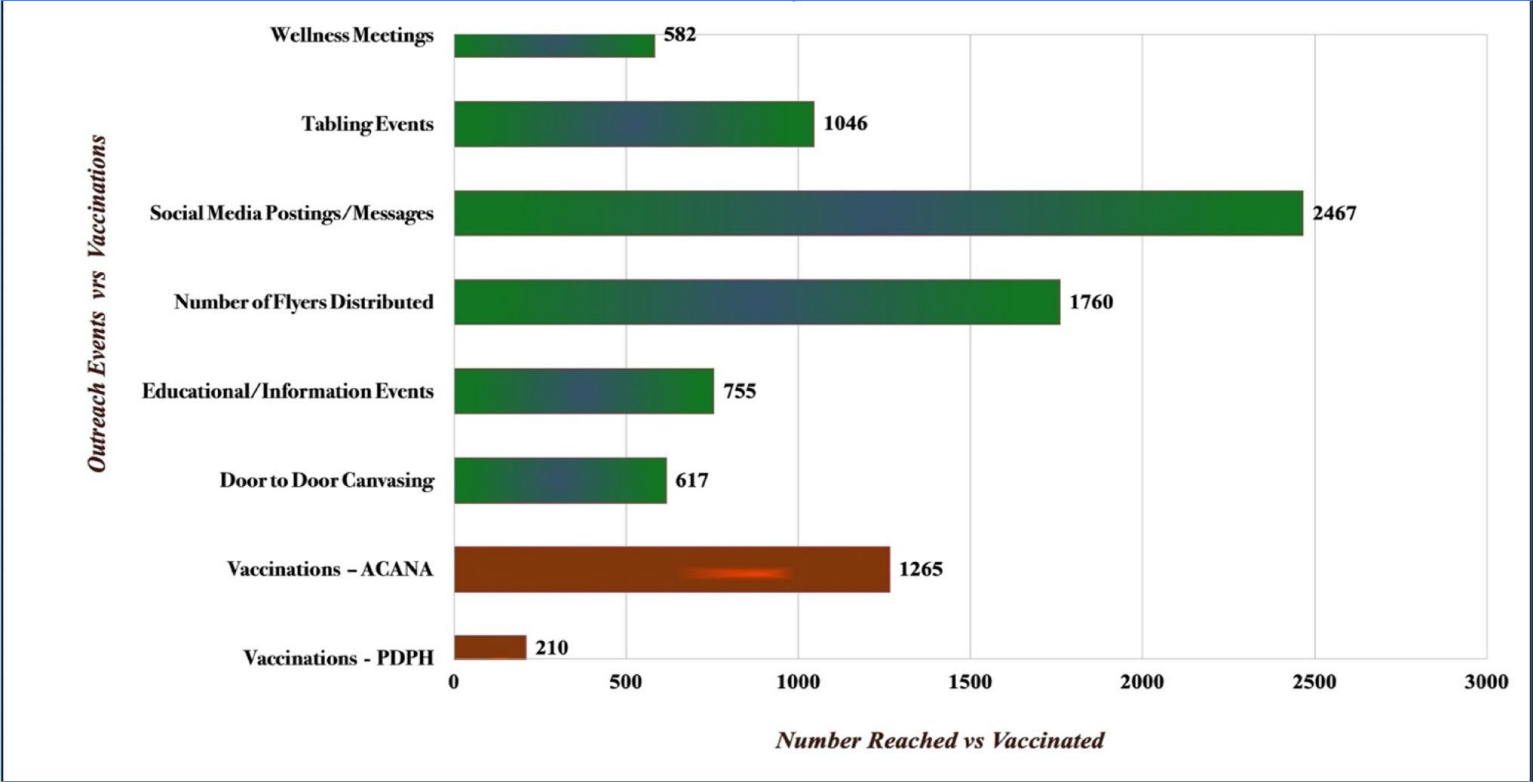
Number vaccinated vs. number reached in community outreach (01/2021–12/2022). Source: ACANA internal data

All available evidence (from ACANA’s reports and event tracking data) showed that community outreach was successful and effective in reaching many African immigrants in the service area. Owing to limitations on in-person contact during the early days of the pandemic, social media postings, text messages, and phone calls were the predominant means of outreach used by health navigators to address community members’ questions/concerns about COVID-19. Close to 3000 members were reached via these media (see Fig. 6). When restrictions eased in mid-2021, ACANA began its face-to-face outreach. It reached 1400 community members during the 52 wellness meetings and health educational workshops it organized. In addition, 35 tabling events were held at churches/mosques and other community locations. These were popular events (1100 people were reached) as they provided a forum for the health navigators to educate community members about COVID-19 and address misinformation. In addition, the health navigators met roughly 600 families during their door-to-door canvassing. Nearly 1800 COVID-19 and other health educational flyers were distributed during all these outreach events (see Fig. 6).

Though the outreach was effective, evidence from ACANA’s records and discussions with health navigators and clinical staff showed that many community members were hesitant about taking the vaccine given the uncertainty and misinformation about its efficacy and safety, as well as inaccessibility of vaccination sites. Cognizant of these logistical barriers and to increase uptake, ACANA opened its vaccine (microsite) clinic in June 2021, in partnership with the Philadelphia Department of Public Health. The first pop-up clinic held at the ACANA microsite vaccinated more than 70 community members. The number vaccinated steadily increased to 1265 by December 2022 (see Fig. 6), an increase that is largely attributable to ACANA’s extensive community-based outreach and broader implementation of vaccine mandates.

**Fig. 7:**
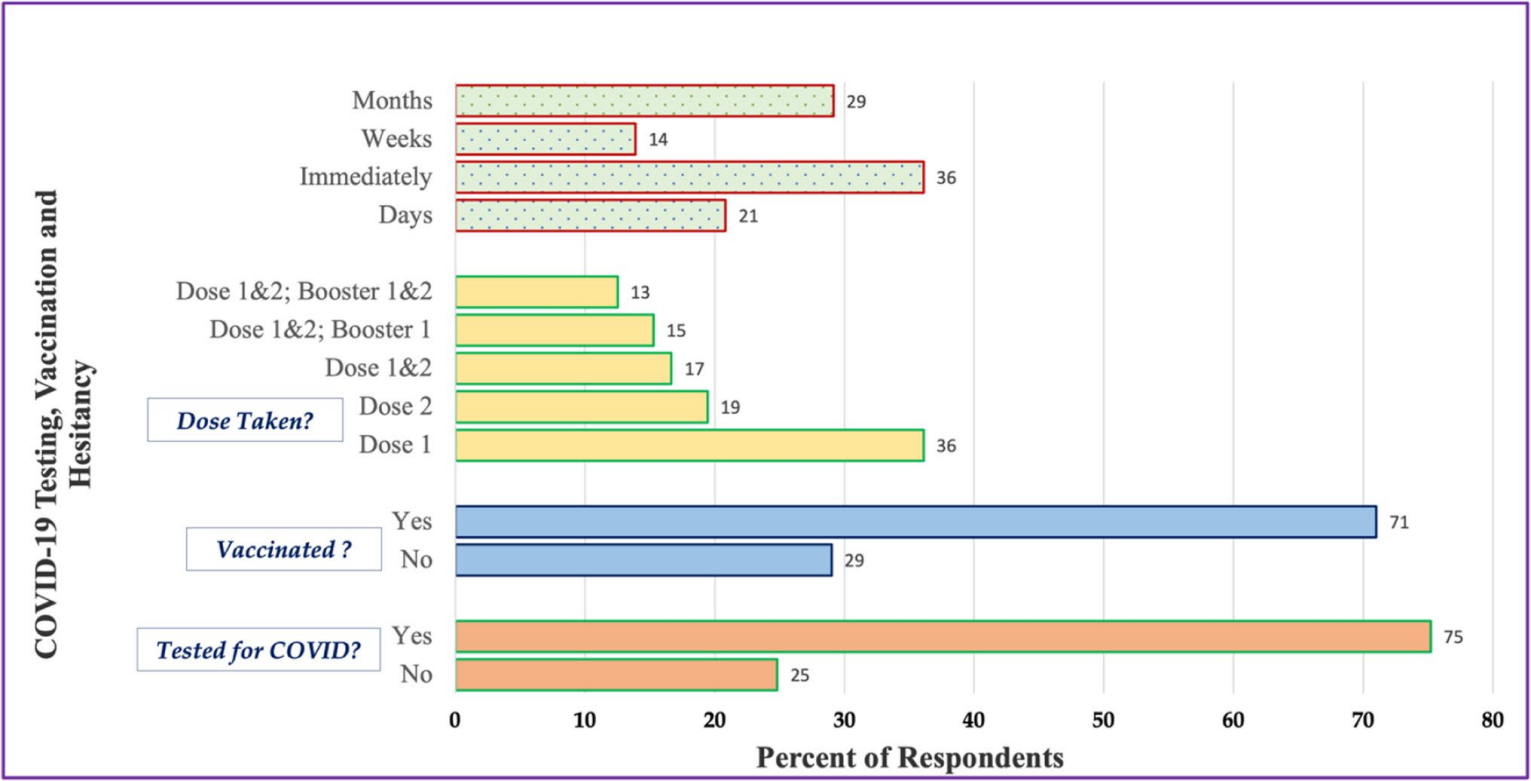
COVID-19 testing and vaccination history, *N* = 100

While ACANA’s outreach was successful in increasing the number of community members vaccinated, internal records and city-wide vaccination data showed a gap in vaccine uptake and persistent hesitancy within black and other minority communities. Though limited in sample size and not fully representative, our random sample survey provided supporting evidence about COVID-19 vaccination rates and hesitancy(see Fig. 7). While 75% of respondents in the survey had tested for COVID, about a third (30%) reported that they had not been vaccinated at the time of the survey. Of those vaccinated, only about half (55%) reported that they had taken either the first or second dose. 57% of our survey respondents took the vaccine either immediately or within days of availability, while close to half (43%) delayed for weeks to months before deciding to take the vaccine, while only about 30% of those survey had taken the full booster vaccines (see Fig. 7).

Study participants explored barriers to vaccine uptake and reasons for hesitancy during interviews, focus group discussions, and responses to open-ended survey questions. Three themes relating to *religious and cultural beliefs*, *misinformation and uncertainty*, and *preexisting barriers to care*, emerged as the bases for COVID-19 hesitancy and uptake.

### Understanding Vaccine Uptake Barriers and Hesitancy

#### Theme 1: COVID-19 Is Rooted in Religious and Cultural Beliefs

There were cultural and religious beliefs about the COVID-19 virus that pre-dated the vaccine rollout and fundamentally shaped participants’ responses to the vaccine campaign. Almost all of them highlighted the significance of religion to COVID-19, specifically the shared belief that the pandemic began as a “judgement” from God and/or the development of the vaccine was a blessing from God. For example, a nurse who was vaccinated and participated in vaccination outreach also discussed her religious beliefs regarding the pandemic’s spread: “I have the belief that this corona was here for months before all of these interventions came in and the slowing down of corona began. The slowing down of corona was not due to the vaccine, it was due to the grace of God. That is how I feel as a person sitting here... I am of the strong belief that corona was judgement.”

Participant’s hesitation must also be framed in the context of their cultural beliefs about illness and health, particularly those relating to the need for preventive health. All participants pointed to how vaccine messaging reflected Western conceptions of illness and not those of other cultures. One clinical staff stated that the framing of COVID-19 vaccine messaging “....that says patients with more chronic diseases will be more critical..” and made people believe that “I am [they are] not ill or elderly, so I [they] will not contract the disease.” Furthermore, the notion of preventive health (taking medications, vaccinations) is still foreign to some participants, despite exposure to conventional medicine [51, 53]. For them, treatments (both conventional and alternative) are only necessary for those who are ill, as one community member explained: “in our [local] knowledge of health and wellness, when you are sick, you take herbs..there is nothing like Western medication. Here, you have to be sick literally to take medicine....when you’re sick you take medicine, when I’m not sick why take it?.” As a result of these beliefs, some found it difficult to understand the necessity or need for vaccines as a preventive measure, particularly when they are asymptomatic. All clinical staff agreed that these beliefs about preventive health were a barrier to vaccination that need to be addressed.

Several clinical staff shared the widely-held beliefs by community members that there were “different injections for Black people versus White people…” and that the vaccine had “...DNA, chips...” that could be (or could become) a nefarious tool to cause infertility, serious illness, or death among Black populations in the US. During focus group discussions and interviews, community members and clinical staff pointed to “that fear of the unknown- past unethical experiments conducted..the Tuskegee Syphilis Study” as a key reason for hesitancy and mistrust. A few survey respondents suggested that urban legends about the imperviousness of African genes to COVID-19 (“that African blood is too strong for COVID”) resulted in lowered risk perception and increased hesitancy to get tested or vaccinated.

#### Theme 2: Hesitancy Is Fueled by Misinformation and Uncertainty

The novelty of the vaccine was a source of concern reported by most participants. They reported that the evolving and sometimes contradictory scientific information accompanying the vaccine rollout fueled further suspicion among community members. A clinical staff reported of one client who refused the get the vaccine because she heard the vaccine is an “alternated vaccine, not a live vaccine, but that they are putting something in you, ...that putting something into you can give you the disease… she can’t understand.” Echoing familiar concerns, one focus group member attributed his hesitancy to uncertainty about the vaccine: “the vaccine information keeps changing ...the confusing part are many variants- Delta and Omicron....the different variants – how many vaccines will be necessary to take?” Underscoring the depth of his suspicion, he concluded that “we need to know the truth about this virus” before he could take it. Other participants linked their uncertainty to “conspiracy theories, myths,” which undermined their trust in the vaccine, and increased their hesitancy, particularly, when “no one took the time to explain the benefits of vaccines nor hear about my[their] fear.”

#### Theme 3: Hesitancy is a Tale of Barriers

Health navigators noted several logistical barriers to vaccine sites. The online registration system for vaccine appointments posed a significant barrier to many community members. One reported that she is not “good with the computer, the internet” and had to rely on her brother to visit and help her register. A clinical staff one of her client’s frustration about the registration system: “At CVS, you have to go on the computer, you can be well-educated, but the computer is a problem- because the computer was not acceptable to us, I remember at Temple we would stand in line to use it. So, the computer, to go online and make the appointment online, that would not have been good at all.” Consequently, clinical staff and health navigators had to go out of their way to visit and help community members without internet access or skills to register for the vaccine. Even if registered, transportation to and from the vaccination sites—which were often located in the inner city, and outside of our participant’s neighborhoods—became a secondary barrier. Older female participants particularly noted the difficulties and the hurdles they had to go through, “walk far to get the bus or trolleys” if they could not get a ride from a family or friend.

In the context of other barriers, English language limitations and low literacy levels made it difficult for many participants to read, understand, or communicate their concerns and uncertainties. Focus group participants generally commented on how the English information and instructions about COVID-19 vaccination made it difficult to feel included and understand the need for the vaccine. Low literacy also compounded language barriers. A clinical staff explained that the vaccine messaging spoke “to the American population, those who can read, very literate, and can understand the consequences [of vaccination] very well”, and that “when it comes to awareness, most of our current immigrants now have elementary level or not much formal education.” These language and literacy limitations further undermined community members’ trust in the vaccine and delayed its uptake.

Legal status and documentation concerns were another barrier to vaccine uptake and a major reason for hesitancy in the African immigrant community. A clinician observed that community members’ hesitancy was linked to the registration process which initially required social security numbers to validate a person’s identity. Although this requirement was quickly relaxed and waived to increase access among marginalized populations, some community members remained concerned about their personal information being recorded on the city’s computer information systems. These concerns were more acute among those who were undocumented and uninsured. One health navigator commented that while vaccines were “accessible” [to the general population], it was “not fully accessible” to Africans and other immigrant groups because the vaccine rollout coincided with “the DACA thing, there’s a huge population that do not have documentation, and they do not want to go there because they are asking for name, social security number, those who don’t have their papers think that they will be reported to the authorities, their names taken, arrested and deported.” Although the vaccine was accessible at community centers and clinics at no charge, many community members reported concerns about their lack of insurance and the cost of the vaccine, because “they don’t have the money to purchase the medicine.”

### Strategies to Increase Vaccine Uptake

Three other sub-themes emerged as strategies to address hesitancy and increase uptake: *the importance of community engagement and mobilization*, *attention to messaging*, and *eradication of barriers to care.*

#### Sub-theme 1: Community Engagement and Mobilization Matters

Community members were satisfied with ACANA’s outreach programs, especially the wellness meetings, tabling events, and health navigators. A participant reported that they decided to take the vaccine after they “attended the biweekly wellness meetings at ACANA, [where] staff help to get me registered for the vaccine online.” During focus group discussions, one community member stated that having tabling events on Chester Avenue and the vaccine clinic “right in our neighborhood at ACANA made it easy … I decided to take the vaccines including the booster. We just walked in, no appointments.” Others echoed similar sentiments and experiences during the discussions.

#### Sub-theme 2: the Medium, the Message, and the Messenger

Community members and health navigators emphasized the importance of using familiar languages and trusted messengers as facilitators in education and messaging about the vaccine. One community member noted that “health navigators explained the benefits of the vaccine … in Mandingo, my language, I understand.” Another commented on the importance of “one-on-one discussions with ACANA staff, their clarifications about COVID, how they addressed their fears and concerns about past unethical experiments.” Community leaders were identified as trusted messengers. Recalling their experiences with Ebola and other epidemics, one clinical staff affirmed the importance of using community leaders and elders in health education: “even in Africa, when they take the vaccines it’s the community who talks to the community to take the vaccines... you see people still question Western medicine, there’s conspiracy theories online... they need people who can relate to them better... speak to them, answer any question.” During in-depth interviews, ACANA clinical staff and health navigators noted that their partnership with community leaders (such as presidents of business associations, pastors, and imams) made it easier to earn the trust of community members and increased vaccine uptake.

Participants’ narratives revealed an overreaching theme that links COVID-19 vaccination to responsibilities beyond the individual. Participants' narratives and responses appealed to a higher order of responsibility and that as global citizens, everyone is responsible for seeing an end to the pandemic. One survey respondent noted: “Everyone should be responsible for their own body. People should not be forced to take vaccines but should make their own decisions.” Several focus group discussants also reiterated the same theme, pointing out that: “The world is a global village and people are social beings. We have a responsibility to protect others..be vaccinated to protect the world...and eradicate the virus.” Most participants noted that the outreach and messaging were more effective when it was delivered, as ACANA staff did in its wellness meetings, using “storytelling and role-play,” health education methods that community members were familiar with from their countries of origin.

#### Sub-theme 3: Removal of Access Barriers

Finally, to ensure successful uptake and decrease hesitancy, participants suggested the removal of several access barriers. The establishment of a vaccine microsite within the community removed one major logistical barrier and was welcomed by community members. Echoing her community’s appreciation for the microsite, a clinical staff stated that: “vaccine clinic came to us, ACANA, the community, it showed that they [the city] actually they care about us...” There was general agreement among focus group participants that the placement of more satellite/pop-up clinics on bus and trolly routes, places of employment, schools, worship, and local pharmacies would make vaccines more accessible to the community. A survey respondent lauded ACANA’s assistance with online vaccine registration systems: “ACANA assisting to register community members online for the PDPH vaccine clinics.....was nice.....internet challenges resolved.” Others suggested that transportation vouchers and other forms of incentives could be provided to participants who completed their doses, in addition to relaxed identification requirements, to increase vaccine uptake.

## Discussion

This study explored the feasibility of a community-based outreach program to increase COVID-19 vaccine uptake among African immigrants in Philadelphia. Increasingly, it has become imperative to deploy such outreach programs because immigrants and other visible minorities tend to be overlooked in traditional modes of health education and outreach [54–57]. Within these populations, community-based organizations (CBOs) have been instrumental in leveraging access to health programs, serving as the cultural and linguistic bridge between members and service providers [29, 38, 40, 41]. However, research examining the feasibility of using such community-based programs to address vaccine uptake in immigrant and refugee populations is limited. A systematic review [58] of vaccine hesitancy and uptake among refugee and immigrants found very few studies [33, 59, 60] that documented the effectiveness of such community-based initiatives in increasing vaccine uptake in immigrant and refugee populations.

The results of ACANA’s outreach showed that community-based programs (including tabling events and tailored messaging) showed promise in decreasing hesitancy and increasing vaccine uptake, and demonstrably more effective in reaching hardest-to-reach populations than mainstream health promotion approaches [33]. ACANA’s outreach program was effective because of its multipronged approach, involving community engagement at different and multiple levels (involvement of community members, local clinics, and communities of faith). The program was anchored around its health navigators who were familiar with the language, culture, and lived experience of community members. Finally, ACANA’s success is also partly dependent on its integration into its neighborhood: its services are within walking distance, with access to pharmacies and small businesses, all of which helped prepare the community and increase its chances for pandemic readiness. The program uniquely demonstrated the effectiveness of community-based initiatives (such as the use of health navigators and vaccine clinics in the community), as well as culturally congruent messaging to address lowered vaccine uptake and hesitancy.

### Recommendations and Lessons for Practice

Based on the success of ACANA’s community-based outreach program, we recommend the following lessons and strategies for decreasing vaccine hesitancy and increasing uptake.

#### Lesson 1: Community Engagement and Mobilization

Community awareness and engagement on all levels (social media, wellness meetings, traditional outreach through partnership with other community-based organizations, businesses, and communities of faith) was pivotal in the success of ACANA’s outreach. ACANA’s promotion of educational events on COVID testing and vaccinations enabled community members to model healthy behavior changes to each other and build trust. Despite its challenges, community engagement was not only more effective in reaching the African immigrant community than traditional means of health promotion, but it also empowered community members to be agents in promoting their health [25, 34, 61].

#### Lesson 2: the Message, the Medium, and the Messenger

A key lesson from this outreach was the importance of framing and messaging about COVID-19. Our participants identified language limitation as a key barrier to their access and utilization of health services and resources. This finding aligns with previous research on language limitations as a barrier to the use of health services [52, 62]. To bridge such a barrier, especially among minority groups experiencing English language limitations, it is vital for health messaging to be delivered by trusted/local messengers in familiar languages and dialects [63–65]. Building on research in Hispanic and Asian communities [66–68], our study showed that direct translation alone is not enough, and that to be responsive, health messaging has to be expressed in plain language to be accessible by community members with low literacy levels.

Given the novelty of COVID-19 and the confusion and misunderstandings reported by our participants regarding the vaccine rollout, it is imperative for health messaging to stress the importance, reasons for, and benefits of vaccines. Messaging must also leverage community citizenship and global responsibility and stress the benefits of the vaccine not only for the individual but the health of their community as well. Research by Kirchoff et al. found that individuals with a sense of collective responsibility (compared to those who did not) had higher odds of COVID-19 vaccine acceptability and getting vaccinated [69].

Besides, how vaccine messaging is packaged and delivered equally matters. Delivery of vaccine information in isolation from other health prevention messaging may not be as effective as if vaccination is seen as part of comprehensive health education and prevention efforts [33, 70]. To increase vaccine uptake, it is imperative to integrate vaccine outreach into other support services (in mental health, depression, HIV, etc.) similar to those offered by ACANA via its wellness meetings, clinics, and other social services. Such one-stop integrated services and messaging have been effective in increasing vaccine knowledge and acceptability [70, 71], as are social media platforms.

During the COVID lockdown, ACANA had to rely on social media platforms to deliver vaccine messaging. Given ACANA’s success and similar initiatives reported elsewhere [72, 73], COVID messaging must leverage the power and efficiency of social media and messaging platforms such as WhatsApp, Instagram, TikTok, and Facebook to extend its reach. Finally, utilizing familiar, well-known trusted messengers (such as health navigators, faith leaders, and Presidents of business associations) is vital to establishing credibility and trust in vaccine education and outreach, thus reducing hesitancy [40, 55, 71]. These health navigators served as trusted and culturally responsive links between ACANA and its community.

#### Lesson 3: Social Determinants of Health Matter

Finally, to ensure successful uptake and decrease hesitancy, participants suggested the removal of logistical access and systemic barriers. The location of vaccination sites, lack of clear information, direction, or convenient transportation to these sites were identified by our participants as major barriers to vaccination uptake and reasons for hesitancy, similar to what has been observed in other minority communities during the COVID-19 pandemic [61, 74, 75]. Lack of health insurance and associated fears of out-of-pocket costs remained barriers to vaccination among our participants, particularly those who were undocumented [58, 61, 76]. Associated fears and apprehension among community members about potential disclosures of their immigration statuses during vaccine registration resulted in hesitancy and lower vaccine uptake [9, 13, 77]. Finally, the computer and Internet literacy skills required by the online registration process reflect the prevalence of the “digital divide” as a determinant of access to health information and resources [78, 79], including COVID-19 [80–82].

ACANA’s free vaccine clinic, accessible to the uninsured, undocumented, and other low-income community members removed a major barrier to access, decreased hesitancy, and increased COVID-19 vaccine uptake. The establishment of a vaccine microsite within the community removed another major logistical barrier (transportation). Given this, the placement of more satellite/pop-up clinics on bus and trolly routes, places of employment, schools, worship, and local pharmacies would make vaccine sites more accessible to diverse communities experiencing transportation barriers [29, 73]. Coupling these efforts with transportation vouchers and incentives to those who completed their vaccine doses will further increase uptake [83, 84].

Despite these contributions, it is worth noting some key limitations of our study. We used a cross-sectional design with a small sample drawn from the Philadelphia region. As a result, our findings may not be generalizable across all African immigrant populations in the US. In addition, given that our participants were recruited through ACANA, there is a likelihood that our findings were skewed toward those already engaged in healthcare services. Future studies must use larger sample sizes and utilize longitudinal designs to explore the dynamics of vaccine hesitancy and uptake. Nevertheless, our study has contributed to emerging knowledge about determinants of vaccine uptake, and uniquely documented the feasibility of multi-pronged community-based initiatives in addressing vaccine hesitancy among African immigrants and similar populations.

## Conclusion

ACANA’s community-based outreach draws on and leverages community social capital as (its members’ unique strengths, talents, and connections) as a key asset. ACANA utilized on-the-ground, person-to-person organization and community advocacy most familiar to community members from their countries of origin. In these ways, ACANA’s outreach helped to build trust and confidence in its messaging, reduced access barriers, and increased vaccine uptake, while advancing health equity through its linkage-to-care programs [85]. In sum, access and availability of vaccines do not automatically translate to acceptability and uptake, given the history of medical mistrust and its concomitant (dis)engagement from health services among Black and other minority communities in the US [86]. Addressing hesitancy among immigrant populations calls for community-based initiatives that draw on the strengths and capabilities of community members due to unique individual and structural barriers they experience.

In light of these findings, we agree with Ogunbajo and Ojikuto’s recent recommendation [34] that vaccine health information campaigns and health providers “explain the benefits in an easy and concise manner, ... providing vaccine-related health information in multiple languages,.... actively engaging religious institutions, social media groups, CBOs, cultural organizations, and key opinion leaders as dissemination avenues for information about the COVID-19...to ensure optimal uptake of the COVID-19 vaccines among first and second generation Black immigrants in the U.S” [34]. Our findings provide evidence that these community-based efforts work in reducing vaccine hesitancy and increasing uptake.

## Supplementary Information

Below is the link to the electronic supplementary material.

Supplementary file1 (DOCX 72 KB)

## Data Availability

De-identified qualitative transcripts and survey data can be made available by contacting the corresponding author.
