# Supplementary material for: Addressing COVID-19 Vaccine Hesitancy and Uptake Among African Immigrants: Lessons from a Community-Based Outreach Program
Source: J Racial Ethn Health Disparities. 2024 Mar 5;12(2):1124–38. doi: 10.1007/s40615-024-01947-9 (PMC11913973; doi:10.1007/s40615-024-01947-9)
Supplement: Supplementary file 1 — Supplementary file1 (DOCX 72 KB) [file 40615_2024_1947_MOESM1_ESM.docx]

**APPENDIX A:**

**SURVEY INSTRUMENT, FOCUS GROUIP AND INDEPTH=INTERVIEW GUIDES**


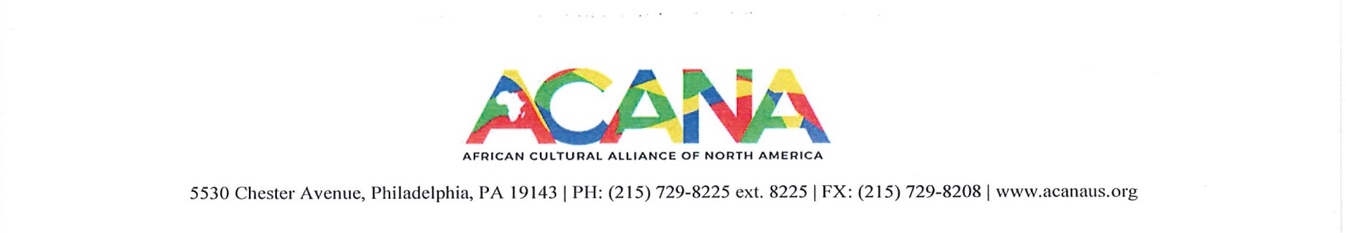


**DETERMINANTS OF VACCINE UPTAKE**

***An Exploratory Study at ACANA, A Non-Profit Organization***

| 1. Age <25 ____ 25-44 ____ 45-64 ____ >64 ____ | |
| --- | --- |
| 1. Gender ___________ | 1. Country of Birth _____________ |
| 1. Religion _____________ |  |
| 1. Years in US <5 ____ 6-10____ 11-20____ >20 ____ | |
| 1. HC Provider (Y/N)? | 1. Have you tested for COVID-19 (Y/N)?________ |
| 1. Have you taken the COVID-19 Vaccine (Y/N)?__________    1. 1stDose (Date/Month/Year) _____________    2. 2nd Dose (Date/Month/Year) _____________    3. 1^st^ Booster (Date/Month/Year) _____________    4. 2^nd^ Booster (Date/Month/Year) _____________ | |
| 1. How long did it take you to decide to take the vaccine? (Immediately, days, weeks, months) | |
| 1. What sources of information (i.e., newspapers, social media, PDPH, CDC, doctors, friends, family, etc.) did you consult when considering the vaccine? | |
| 1. Did you find it difficult to get vaccinated? If so, why? | |
| 1. What would have made it easier for you to get the vaccine? | |
|  | |
| ***CDC: Centers for Disease Control. PDPH: Philadelphia Department of Public Health*** | |


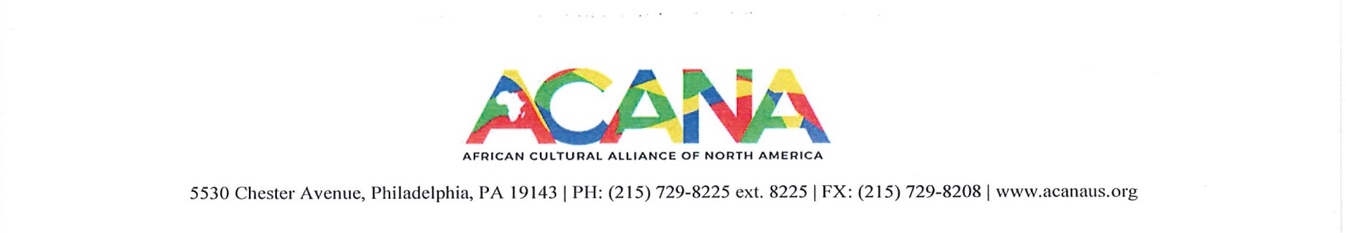


**COMMUNITY FOCUS GROUPS**

***Conversations on Mental Health Literacy and COVID-19 Vaccine***

1. What are your views on COVID-19 Vaccine?
2. What would have made it easier for you and your community to get the vaccine?
3. What are the challenges for getting access to and receiving the vaccine?
4. Were you hesitant initial to take the vaccine? Y/N, Why?
5. Do you know someone who was hesitant to get the vaccine? What made them change their mind?


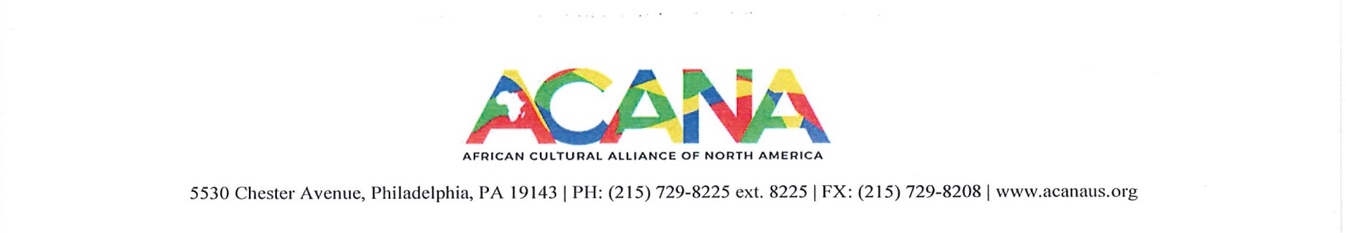


**HEALTH NAVIGATOR FOCUS GROUPS**

***Experiences of Health Literacy and COVID-19 Vaccine***

1. What are your views on COVID-19? COVID-19 Vaccine?
2. What do community members believe about COVID? What have you heard or experienced?
3. What strategies have worked to address COVID knowledge, health literacy?
4. What would have made it easier for you and the community to get the vaccine?
5. What are the challenges for getting access to and getting vaccine?
6. Why are some of the barriers you experienced in getting community members vaccinated?.
7. In general, what worked to encourage community members to take the vaccine?
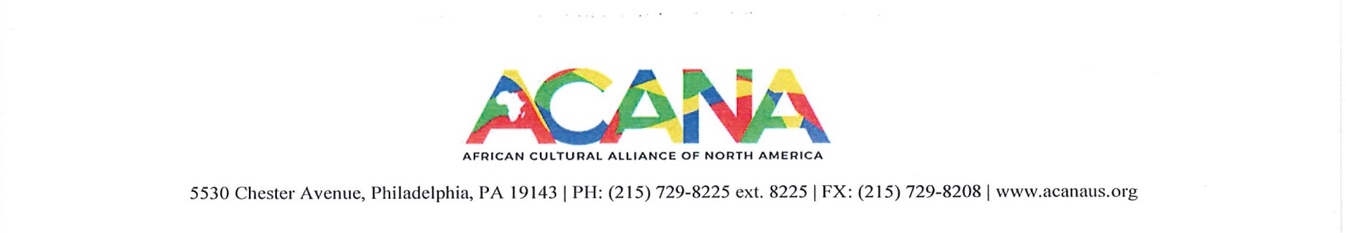


**IN-DEPTH INTERVIEWS WITH CLINICAL STAFF**

***Experiences of Health Literacy and COVID-19 Vaccine***

1. What are your thoughts on the COVId-19 vaccine campaign for African immigrants in Philadelphia?
2. Do you think the vaccine was accessible to African immigrants in Philadelphia?
3. What could have been improved regarding the city's COVID-19 response?
4. What were some of the reasons for hesitancy you encountered in your work?
5. What did you think of the messaging that was targeting African immigrants to encourage vaccine uptake?
